# Identifying Subgroups of Patients With Autism by Gene Expression Profiles Using Machine Learning Algorithms

**DOI:** 10.3389/fpsyt.2021.637022

**Published:** 2021-05-12

**Authors:** Ping-I Lin, Mohammad Ali Moni, Susan Shur-Fen Gau, Valsamma Eapen

**Affiliations:** ^1^School of Psychiatry, The University of New South Wales, Sydney, NSW, Australia; ^2^South Western Sydney Local Health District, Liverpool, NSW, Australia; ^3^Department of Psychiatry, National Taiwan University Hospital and College of Medicine, Taipei, Taiwan

**Keywords:** autism spectrum disorder, genomics, social cognition, language, machine learning

## Abstract

**Objectives:** The identification of subgroups of autism spectrum disorder (ASD) may partially remedy the problems of clinical heterogeneity to facilitate the improvement of clinical management. The current study aims to use machine learning algorithms to analyze microarray data to identify clusters with relatively homogeneous clinical features.

**Methods:** The whole-genome gene expression microarray data were used to predict communication quotient (SCQ) scores against all probes to select differential expression regions (DERs). Gene set enrichment analysis was performed for DERs with a fold-change >2 to identify hub pathways that play a role in the severity of social communication deficits inherent to ASD. We then used two machine learning methods, random forest classification (RF) and support vector machine (SVM), to identify two clusters using DERs. Finally, we evaluated how accurately the clusters predicted language impairment.

**Results:** A total of 191 DERs were initially identified, and 54 of them with a fold-change >2 were selected for the pathway analysis. Cholesterol biosynthesis and metabolisms pathways appear to act as hubs that connect other trait-associated pathways to influence the severity of social communication deficits inherent to ASD. Both RF and SVM algorithms can yield a classification accuracy level >90% when all 191 DERs were analyzed. The ASD subtypes defined by the presence of language impairment, a strong indicator for prognosis, can be predicted by transcriptomic profiles associated with social communication deficits and cholesterol biosynthesis and metabolism.

**Conclusion:** The results suggest that both RF and SVM are acceptable options for machine learning algorithms to identify AD subgroups characterized by clinical homogeneity related to prognosis.

## Introduction

Clinical heterogeneity is a norm rather than an exception in autism spectrum disorder (ASD), a complex neurodevelopmental disorder characterized by social communication deficits and stereotyped behaviors. Heterogeneous clinical features pose great challenges for diagnostics for ASD, such that children who receive a diagnosis of ASD have a range of vastly different presentations, trajectories, and outcomes. Further, the diagnostic criteria for ASD have been continuously revised through different editions of the Diagnostic and Statistical Manual for Mental Disorders (DSM), particularly the substantial changes in the 5th edition (DSM 5) where the wide range of clinical presentations have been brought together under a single ASD diagnostic entity (1). The current diagnostic system lacks an evidence-based approach and we urgently require a scientific approach to understanding which interventions are likely to be the most effective for which child with ASD (2). Accumulating evidence has shown that no pharmaceutical treatments have thus far been conclusively found to substantially reduce core symptoms of ASD (3). This may be partially attributable to the fact that most clinical trials did not take clinical heterogeneity into account and hence treatment effects remain equivocal. Variable clinical presentations may reflect different biological pathways. The identification of biomarkers for etiological pathways may hence hold the key to unraveling mechanisms underlying the variation in clinical presentations (4), which in turn may pave the way for personalized medicine in ASD.

The goal of identifying biomarkers for clinical homogeneity is to tackle challenges arising from clinical heterogeneity for research on either etiologies or treatments of ASD. One of the most extensively studied biomarkers for ASD is genetic factors. There are two different strategies to evaluate genetic markers for clinical heterogeneity: bottom-up and top-down approaches. The bottom-up approach is to define a priori subgroups using phenotypic information under the premise that some genetic loci are more likely to contribute to susceptibility to disease in a certain subgroup(s). Therefore, stratifying the population by a clinical marker (e.g., age of onset) will allow investigators to detect genetic association effects that are larger in certain subgroups. The top-down approach, on the other hand, is based on the premise that certain genetic markers can be used to distinguish subgroups, each of which is characterized by relatively homogeneous phenotypic profiles underscored by similar biological pathways—which imply similar therapeutic targets. Many of the earlier genome-wide linkage or association studies that aimed to unravel genetic underpinnings of clinical heterogeneity chose the second approach, which is to identify genetic markers associated with the phenotype defined by strict diagnostic criteria of ASD (5–7). Using the data from the Autism Diagnostic Interview-Revised (ADI-R) (8), Autism Diagnostic Observation Schedule (ADOS) (9), Vineland Adaptive Behavior Scales (VABS) (10), head circumferences, and ages at assessment as classifying variables, Veatch et al. identified clinically similar subgroups of individuals with ASD and found that the genotypes were more similar within subgroups compared to the whole population—the proportion of the total genetic variance contained in a subpopulation was 0.17 (11). However, this approach has not yielded highly replicable and clinically meaningful findings that can lead to conclusively validated etiological factors yet (12). Furthermore, another genome-wide association study of 2,576 families with ASD probands did not discover any genetic loci that exert a larger effect on the disease risk in subpopulations defined by the diagnosis, IQ, and symptom profiles; heritability estimates were also found to be similar in subpopulations to the whole population (13). Results from different groups show that an increased number of gene-truncating variants (highly pathogenic variants) may exert a considerable impact on IQ in ASD patients (14, 15); and higher burden of this pool of variants in ASD patients correlates with lower IQ scores. These studies showed that genomic approaches are able to identify genetic loci exerting larger effect on disease risk or associated with clinical outcomes, although genetic loci must be considered in an additive manner.

The top-down approach often starts with a few selected genetic loci associated with the disease. Despite fruitful findings from genome-wide and candidate gene-based association studies, few genetic loci can be used to improve accuracy in diagnostics or optimize treatment effects of therapeutics for ASD. Nevertheless, several genetic markers are found to be useful for classifying patients with ASD into relatively homogeneous subgroups. For example, Bruining et al. reported prominently higher symptom homogeneity in both the ASD group with 22q11 deletions and ASD group with Klinefelter Syndrome (KS), compared to the heterogeneous ASD sample (16). Transcriptomic profiles have also been used to identify genetic markers to classify individuals with ASD. Hu and Lai used the gene expression data to identify a subset of the “classifier” genes, which resulted in an overall class prediction accuracy of nearly 82%, ~90% sensitivity, and 75% specificity (17). These results seem to demonstrate the value of the top-down approach.

Determining subgroups of ASD is challenging mainly because of the complexity of biological factors and clinical heterogeneity inherent to ASD. To tackle these challenges, one of the solutions is to implement state-of-the-art statistical methods that can efficiently parse through high-dimensionality data, such as machine learning (ML) algorithms, to differentiate subgroups with meaningful etiological, diagnostic, or therapeutic implications (18). Previous evidence suggests that ML algorithms can be used to reduce the number of items from standardized ASD assessment tools to make the assessment more efficient (19) and predict clinical outcomes with ASD phenotypic clusters and genetic data of copy number variations (20). The ML algorithms appear to be useful to identify phenotypic clusters as ASD subgroups that can predict clinical outcomes (21). In the current study, we attempted to implement the ML algorithms in the context of the bottom-up approach, which is to identify clusters using genomic information, and then explore the relationship between the genomic clusters and clinical features of ASD.

## Methods

### Data Collection

The goal of the current study is to evaluate whether transcriptomic profiles correlated with clinical severity levels of ASD—which were measured with social communication questionnaire (SCQ) (22), can classify patients into two subgroups defined on the basis of language (i.e., the subgroup with language impairment vs. the subgroup without language impairment). The language function is considered as a strong predictor for cognitive ability and adaptive skills in children with ASD (23), and its variation within ASD patients is influenced by genetic factors (24–26). The presence of language impairment was defined as the total score (verbal) >10 in the section of Qualitative Abnormalities in Communication in Autism Diagnostic Interview-Revised (ADI-R) (27). A total of 31 children diagnosed with ASD were recruited in the current study. The clinical diagnoses were made by Gau, a board-certified child psychiatrist, and confirmed by the ADI-R interview with the parents. The Chinese version of the ADI-R been approved by the Western Psychological Services in May 2007 (28) mRNA was extracted from lymphoblastoid cell lines (LCL) of all participants. The microarray experiment was performed at the Core Laboratory of National Taiwan University Hospital in Taiwan, using the Affymetrix Human Genome U133 Plus 2.0 Array (Affymetrix Inc., Santa Clara, CA, USA). The experimental procedures followed the protocols provided by the manufacturer. The study was conducted with the ethical approval by the Institutional Research Board at National Taiwan University Hospital in Taiwan.

### Statistical Methods

#### Transcriptome-Wide Association Analysis

We evaluated the integrity of 28S and 18S rRNA by electrophoresis of 2 mg of total RNA in 1.2% agarose gel containing 2.2 M formaldehyde and in a running buffer containing 0.2 M of MOPS (pH 7.0), 20 mM of sodium acetate and 10 mM of EDTA (pH 8.0). The A260/A280 ratio was used to measure the quality of RNA. The ratio between 1.9 and 2.1 was considered good quality. The intensity files of all the subjects were input into the computer program GAP: Generalized Association Plots (29, 30) for quality control using visualization and descriptive statistics. We used the Robust Multi-array Analysis (RMA) method to normalize the data (31). In order to filter out probe sets with low variations and to reduce the impact of multiple comparisons, we kept only the 1,000 probe sets with the largest standard deviations. We searched for differential expression regions (DERs) by prioritizing the gene expression levels associated with the clinical severity indicated by SCQ scores, we used the generalized linear model to screen for probes across the whole genome with mRNA levels associated with the SCQ scores with unadjusted *p* < 0.00001. All original intensity ratio data were transformed into logarithmic 2 values after being normalized. We controlled for the batch effect by adjusting for the batch as a binary covariate since there were two batches. These probes constitute the primary source of predictors to determine ASD subgroups.

#### Gene Ontology and Pathway Analysis

The DERs with a fold-change >2 were selected for the gene ontology and pathway analysis to evaluate the biological relevance and functional pathways of the significant genes. We have incorporated the KEGG (32), WikiPathways (33), BioCarta (34), and Reactome (35) pathway database for the cell signaling pathways. We have also considered the GO Biological Process (2018) database for gene ontological analysis (36). The GO terms and pathways enriched by the list of genes were identified using the hypergeometric analyses with an adjusted *P* ≤ 0.05 was considered as statistically significant.

#### Gene Over-Representation Analysis

Then we used the webtool at ConsensusPathDB (http://cpdb.molgen.mpg.de/) to identify pathway-pathway interaction network (CPDB analysis) (37). The analysis criteria included: (1) one-next neighbors for the radius with *p* < 0.01, (2) pathway-based sets at least two overlapped genes and *p* < 0.01, and (3) gene ontology level 2 categories with *p* < 0.01. The results from the second approach helped visualize the possible “hub” pathway from the top 10 networks associated with the candidate genes.

We chose two machine learning (ML) algorithms to evaluate the clustering results: random forest classification and support vector machine algorithms. The presence of language impairment was considered as a dichotomous clinical outcome to determine classification errors. We chose the first ML algorithm proposed by Shi and Horvath (38). We used the Random Forest classification (RF) algorithm in an unsupervised mode to generate a proximity matrix. The gene expression data were analyzed using RF using two different approaches for comparison. The first approach is to reduce data dimensionality using principal component analysis to identify principal component (PC) scores for each subject. The top 10 PCs were selected to calculate the proximity matrix that provides a rough estimate of the distance between samples based on the proportion of times the samples end up in the same leaf node. The proximity matrix values were then converted to a dissimilarity matrix to classify the sample into two subgroups using partitioning around medoid (PAM) (39). The second approach is to use the information of all 191 probes with gene expression levels significantly associated with SCQ scores to generate the RF proximity matrix. Similarly, the RF proximity matrix was used to classify the sample into two subgroups using the PAM clustering analysis (39) to classify the patients into two clusters to determine the final cluster assignment. The RF-PAM clustering analysis could allow us to evaluate the classification error by calculating the frequency of patients with language impairment in the cluster, in which the majority of patients had no language impairment, and vice versa.

We further chose Support Vector Machine (SVM) as the second ML algorithm to classify the patients into two subgroups (40). To reduce data dimensionality, we implemented principal component analysis to identify the principal component (PC) scores for each subject. The data of PC scores were split in a 7:3 ratio—in other words, 70% of the data was used for training the model and the remaining 30% was for testing the model. Estimating the C (Cost) parameter to classify the data was performed using SVM with the linear kernel function. The choice of kernel function was made based on the recommendation from a prior study that using microarray data to predict the diagnosis of colon cancer—which concludes that linear kernel function leads to a lower prediction error than the RBF, quadratic, and polynomial kernel functions (41). The prediction accuracy and Kappa value estimated when the C value was held constant at 1. The Kappa value was calculated using the formula (*p*_o_ – *p*_e_)/(1-*p*_e_), where *p*_o_ and *p*_e_ denote the observed agreement and expected agreement for classification, respectively. We further used the confusion matrix, which contains the number of correct and incorrect predictions summarized with count values and broken down by each class, to predict the prediction accuracy of the SVM model. The accuracy is calculated as (TP + TN)/(TP+TN+FP+FN), where TP and TN refer to true positives and true negatives, respectively; FP and FN refer to false positives and false negatives, respectively. These two measures (i.e., accuracy and Kappa value) were chose to evaluate the SVM performance as recommended by previous studies (42, 43). The Kappa statistics could lead to a biased performance estimate in unbalanced situations (44), which is not the characteristic of the current sample. The SVM analysis was performed using the R package “*caret*” (45).

## Results

**Figure 1:**
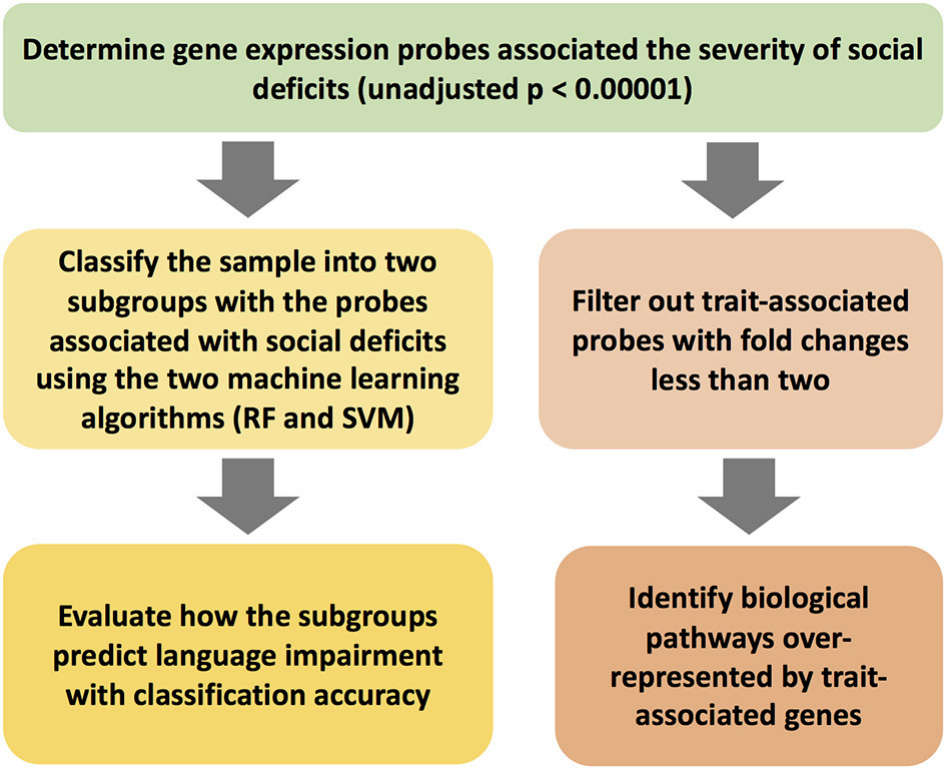
The workflow of the study scheme.

The workflow of the current project is shown in Figure 1. The clinical features of the 31 subjects are summarized in Table 1. The group with language impairment and the group without language impairment has significant differences in clinical features associated with both social communication function and verbal IQ scores.

**Table 1:** Clinical features of the patients in the current study.

	**Language impairment (51.3%)**	**No language impairment (48.7%)**	Relationship with language impairment^*^
Age	9.00 (*SD*: 2.52)	8.91 (*SD*: 3.99)	*P* > 0.05
ADIR-BV	17.83 (*SD*: 3.27)	8.55 (*SD*: 1.13)	*P* < 0.0001
ADIR-BN	8.92 (*SD*: 2.71)	3.64 (*SD*: 1.43)	*P* < 0.0001
SCQ	22.19 (*SD*: 4.84)	11.47 (*SD*: 4.84)	*P* < 0.0001
VIQ	82.08 (*SD*: 20.77)	111.91 (*SD*: 10.12)	*P* = 0.0003
PIQ	90.83 (*SD*: 15.74)	101.36 (*SD*: 15.34)	*P* > 0.05
SRS	89.61 (*SD*: 16.12)	79.55 (*SD*: 27.99)	*P* > 0.05

*ADIR-BV, Autism Diagnostic Interview–Revised, Qualitative Abnormalities in Communication, Total Verbal score. ADIR-BN, Autism Diagnostic Interview–Revised, Qualitative Abnormalities in Communication, Total Non-Verbal score. SCQ, Social Communication Questionnaire score; VIQ, verbal IQ; PIQ, performance IQ; SRS, Social Responsiveness Scale score*.

* *The student's t-test was performed to evaluate whether the the two subgroups classified by the presence of language impairment had different values in each continuous variable*.

**Figure 2:**
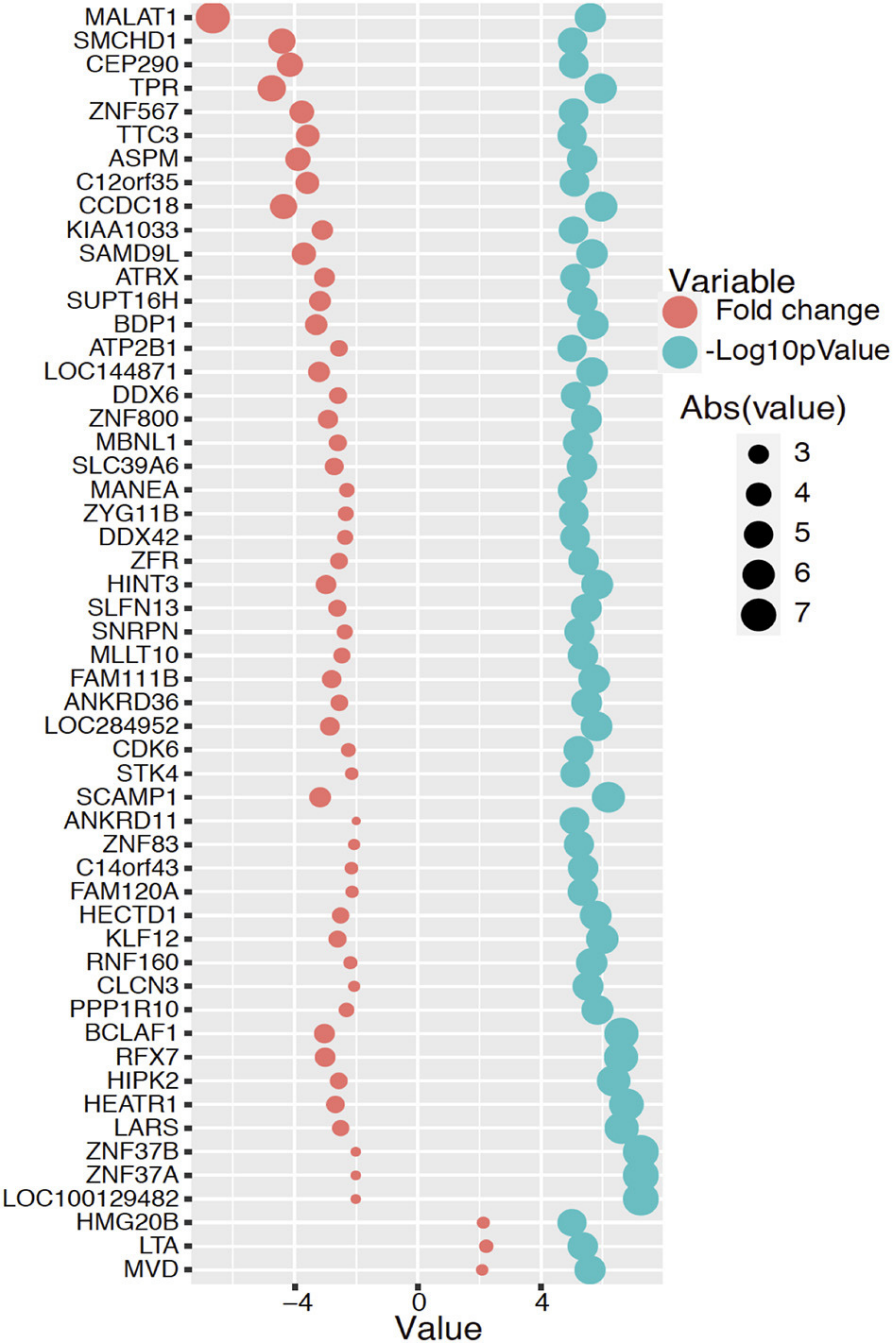
Differentially expressed 54 genes with fold changes and –logarithmic 10 adjusted *p*-values. The red circle represents logarithmic fold change and the blue color circle represents –logarithmic 10 adjusted *p*-value for each significant gene.

**Figure 3:**
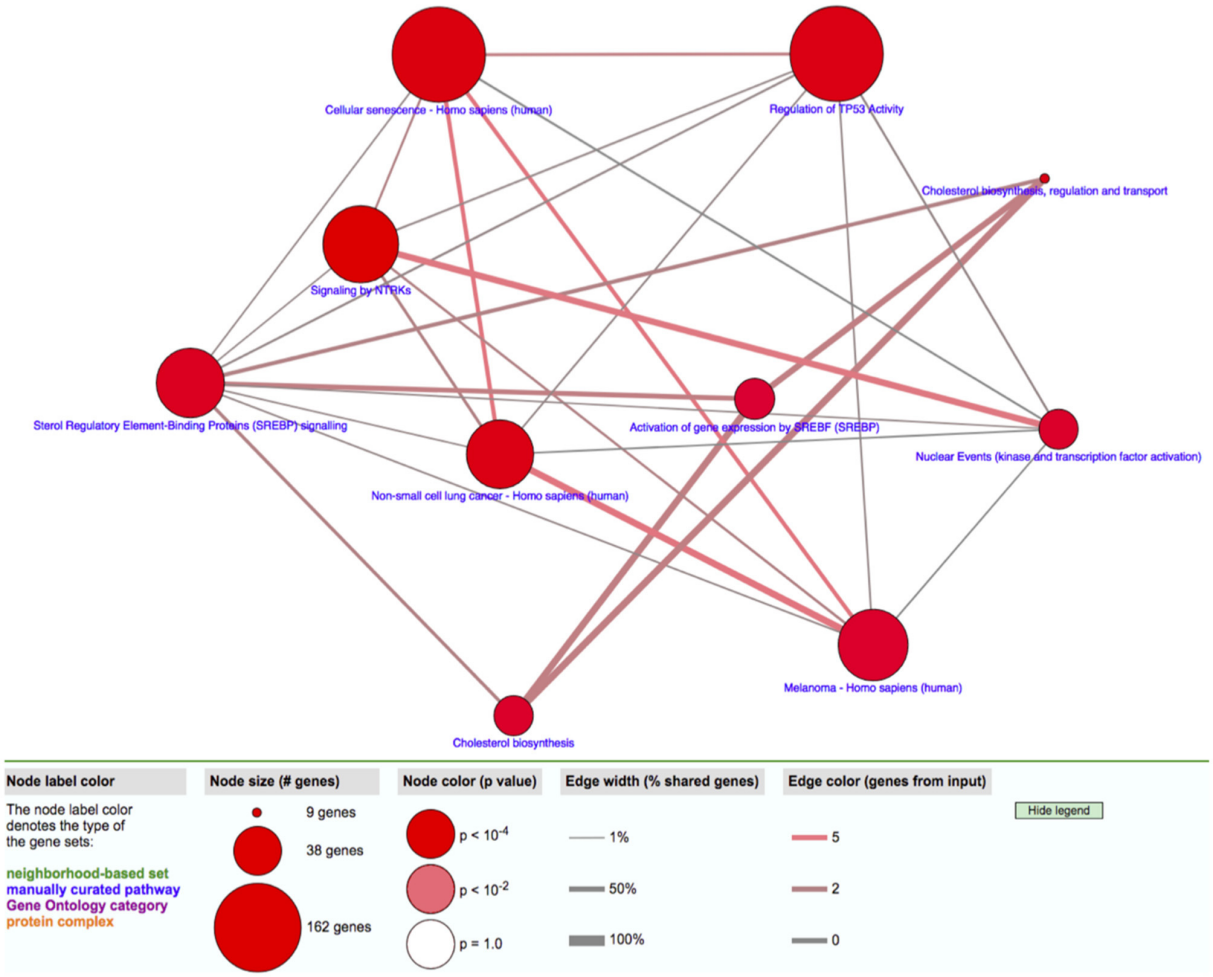
Gene network analysis. The relationship among pathways enriched with candidate genes with expression levels associated with SCQ scores is shown.

The transcriptomic association study reveals 191 probes that were statistically significantly associated with SCQ scores with a *p* < 0.00001. The batch effect seemingly did not affect the association results (Supplementary Figure 1). We selected 54 of them with a fold-change >2 for the pathway analysis. Differentially expressed 54 genes with logarithmic fold changes and –logarithmic 10 adjusted *p*-values are listed in Figure 2. Only three pathways were found to be over-represented by these 54 genes with adjusted *p* < 0.05: cholesterol biosynthetic process (GO:0006695), secondary alcohol biosynthetic process (GO:1902653), and regulation of signal transduction by p53 class mediator (GO:1901796). The CPBD analysis shows that Sterol Regulatory Element-Binding Proteins (SREBP) signaling pathway is the pathway connected with 9 of the 10 pathways including cholesterol biosynthetic pathway, so it can be regarded as the “hub” associated with genetic network for ASD (Figure 3). This pathway of SREBP focuses on the regulation of lipid metabolism by SREBP.

**Figure 4:**
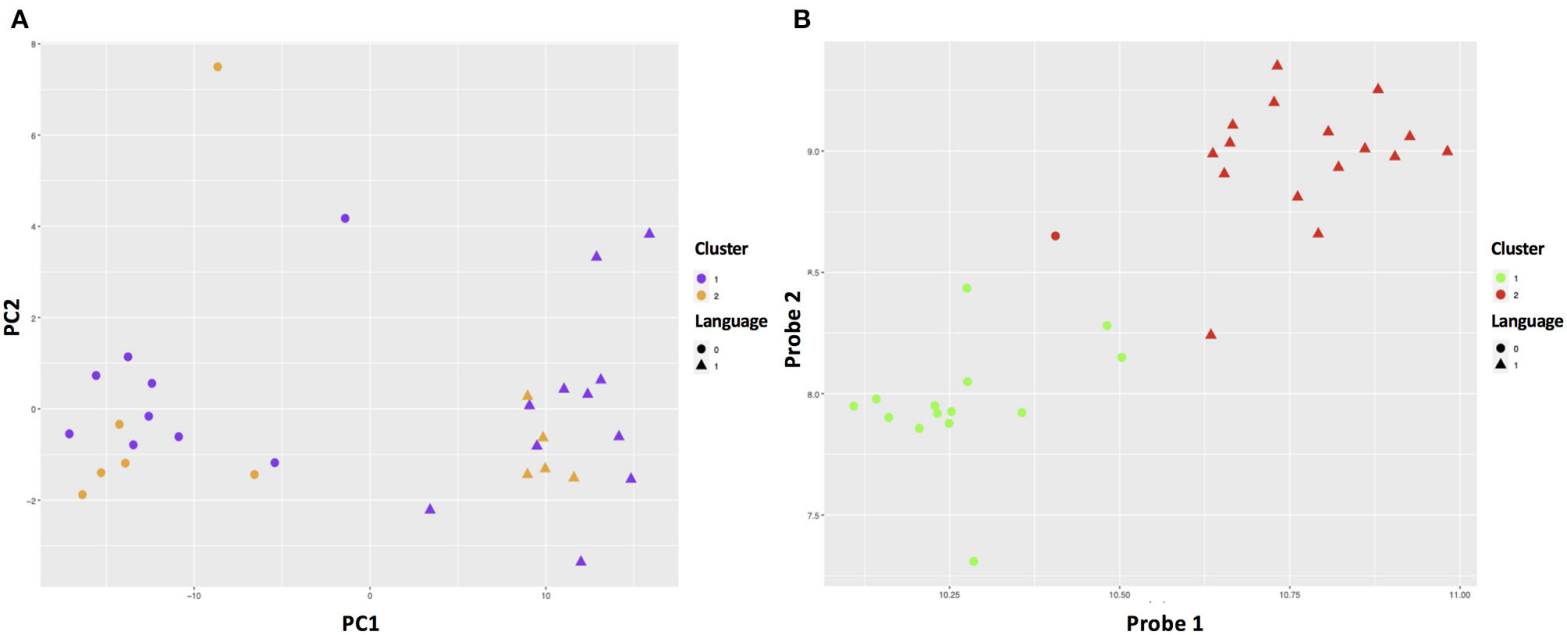
ASD subgroups identified using RF and PAM clustering algorithms. Dim1 and Dim2 correspond to principal components 1 and 2, respectively. **(A,B)** The results based on the top 10 principal components (PCs) and the 191 probes, respectively. We used the first two predictors to make the plots to demonstrate how different approaches classified the sample.

**Figure 5:**
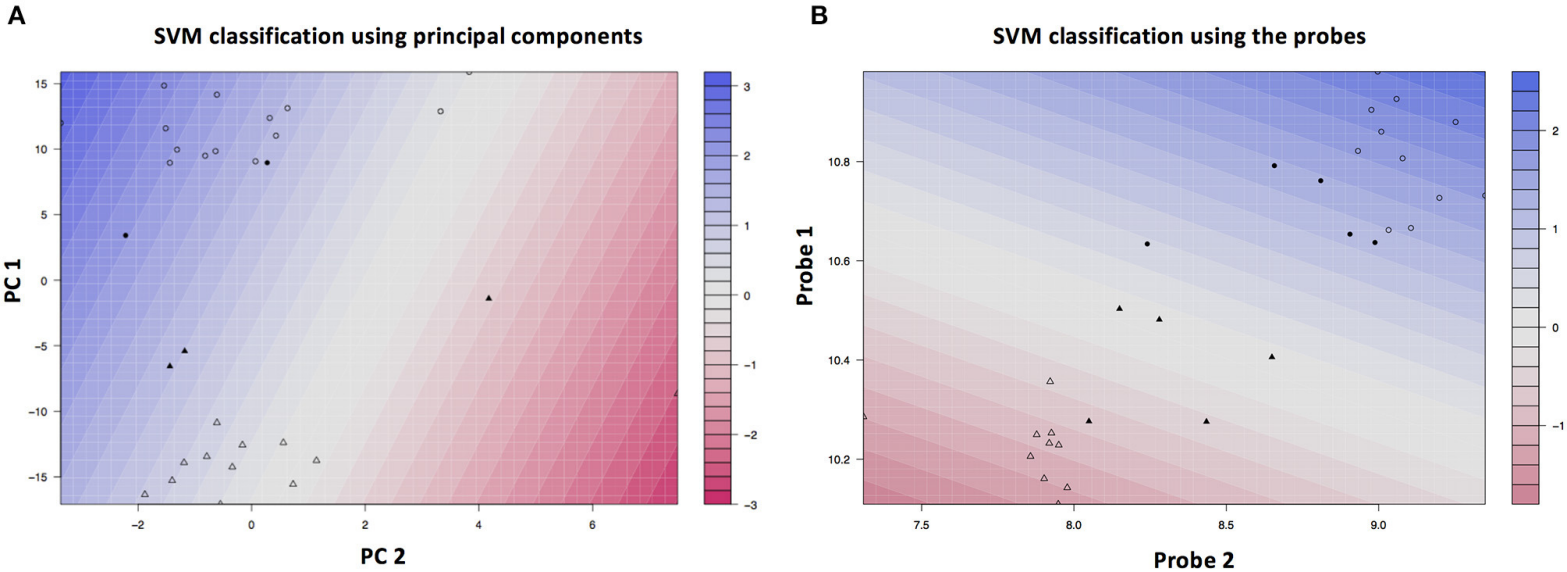
SVM clustering results based on the top PCs. **(A)** Shows the color gradient that indicates how confidently a new point would be classified based on its features. PC1 and PC2 represent the first and second principal components, respectively. **(B)** Shows the color gradient that indicates how confidently a new point would be classified based on its features when predictors were based on all SCQ-associated probes. Probe 1 and probe 2 represent the first and second probes, respectively. The solid symbols indicate the support vectors and the hollow circles indicates other subjects. The circles and triangles represent the first and second subgroups, respectively.

The RF-PAM analysis identified two clusters (Figure 4). The classification accuracy was 67.7% when the top 10 PCs were used to generate the proximity matrix, while the classification accuracy was 96.9% when all 191 probes were used to generate the proximity matrix. The SVM analysis based on the top 10 PC scores shows that the clustering results reached classification accuracy at 93.3% (95% CI 68.1–99.8%) and no-information rate (i.e., the largest proportion of the observed classes) at 53.3% (*p* = 0.0011). Other parameters relevant to prediction performance include Kappa value = 0.86, sensitivity = 0.86, specificity = 1.00, and balanced accuracy = 0.93. The SVM analysis using the information of all probes with differential gene expressions associated with SCQ scores yielded a slightly higher classification accuracy than the SVM analysis based on the top 10 PC scores. The classification accuracy at 99.9% (95% CI 78.2–100%) and no-information rate (i.e., the largest proportion of the observed classes) at 53.3% (*p* = 8.035 × 10^−5^) were achieved when 191 probes were analyzed. This classification accuracy can be demonstrated in gene expression level distributions stratified by language impairment (Supplementary Figure 2). The SVM clustering results are shown in Figure 5. The results suggest that the first two principal components could identify support vectors that fell in the area with better prediction confidence (Figure 5A), compared with the results predicted by individual probes (Figure 5B). The predicting performance of the RF-PAM and SVM algorithms is listed in Table 2.

**Table 2:** Predicting performance of two machine learning algorithms.

**Method**	**Predictors**	**Prediction accuracy**
RF-PAM	191 probes	96.90%
RF-PAM	10 PC^*^	67.70%
SVM	191 probes	99.90%
SVM	10 PC^*^	93.30%

* *Principal component*.

## Discussions

We conducted a proof-of-concept study to demonstrate how transcriptomic data from a small sample could provide useful biomarkers to classify ASD subgroups. The selection of the predictors was based on DERs associated with SCQ scores, which indicate the variation in severity levels of social communication deficits, a hallmark clinical feature of ASD. The DER with strongest evidence for the association with social deficits in our sample is matched with the HEATR1 gene (HEAT Repeat Containing 1). The HEART1 gene is associated with schizophrenia (46). The HEATR1 gene abnormalities in the brain during the embryonic stage has been reported in zebrafish (47). The candidate genes that harbor these DERs suggest several genetic pathways that modulate the variation in social communication functions. Among these pathways, the pathway of cholesterol biosynthesis/metabolism and sterol regulatory element-binding proteins (SREBP) pathway—cholesterol metabolism appear to act as hubs that connect other top SCQ-associated pathways. Particularly, the SREBP pathway shares most genes with other SCQ-associated pathways. These two pathways are related to lipid metabolism. Cholesterol synthesis and uptake are tightly modulated at the transcriptional level through negative feedback control, which is regulated by SREBPs (48). The relationship between lipid metabolism and brain functions has been well-documented. A growing body of evidence has indicated that cholesterol metabolism plays a key role in synaptic functions (49–51). Dysregulated cholesterol metabolism has been extensively documented in ASD (51–58). A recent study implemented a personalized medicine approach combining healthcare claims, electronic health records, familial whole-exome sequences, and neurodevelopmental gene expression patterns, and identified an ASD subtype characterized by dyslipidemia (59). There are certainly several other genetic pathways involved in molecular mechanisms underlying social communication deficits. Nevertheless, our results indicate that cholesterol synthesis/metabolism pathways act as hubs that connect most other biological pathways, which suggest that the genomic functional changes associated with lipid metabolism may moderate other genomic changes, such as the p53 signaling pathway, that regulate social communication functions.

Using the DERs as biomarkers, we clustered the sample into two subgroups using two different ML algorithms. Both the RF-PAM and SVM analyses yielded similar levels of classification accuracy when all 191 markers were utilized. However, compared to the analysis using the RF-PAM algorithm, the analysis using the SVM algorithm seemed to be more robust when we performed dimension reduction for all the 191 markers with the PCA method. The RF algorithm is applicable when there are more predictors than observations, relatively insensitive to the noise (e.g., a large number of irrelevant genes), and does not rely on excessive fine-tuning of parameters (60). RF algorithm is more robust to small sample size as the SVM algorithm (61, 62). However, Brown et al. found that SVM outperforms other techniques that include Fisher's linear discriminant, Parzen window, and tow decision tree learners when using gene expression data to predict clinical outcomes (63). Additionally, Statnikov et al. conducted a comprehensive comparison of RF and SVM using microarray data for 22 diagnostic and prognostic datasets and concluded that SVM is superior to RF in terms of classification accuracy (64). Although the purpose of this study is not to comprehensively evaluate which ML algorithm outperforms the other ML algorithm, our results seem to lend some support to the robustness of the SVM algorithm. Nevertheless, the RF algorithm is at least as robust as the SVM algorithm when the dimension of input variables is not substantially reduced.

One of the major limitation of the current study is the small sample size. Nevertheless, some machine learning algorithm, such as SVM, can handle a small sample with a large number of features. Additionally, model overfitting may arise due to a lack of another independent sample for validation. Furthermore, unknown confounders may cause spurious associations between the phenotype and genomic markers. However, the goal of this proof-of-concept study is prediction of subtypes rather than the identification of etiologies. Therefore, confounders would not yield a substantial impact on prediction results (65).

The clinical and etiological heterogeneity in ASD has meant that there is considerable variability in treatment outcomes across different interventions and between individuals receiving the same intervention. Hence the traditional diagnostic and “one size fits all” approach to ASD intervention needs improvement. Further, we currently do not have a sufficient understanding of “what would work for whom,” thereby limiting opportunities for maximizing outcomes for children and their families with economic ramifications for broader society. In this context, ML algorithms have been found to be useful in predicting diagnostic accuracy in ASD with neuroimaging data (66). Further, one recent study used Gaussian Mixture Models and Hierarchical Agglomerative Clustering, which provide a statistical framework for learning latent cluster memberships to determine ASD subgroups with differentiated treatment responses (67). Our findings that using ML algorithms, children could be classified into two groups based on the presence of language impairment, offers promise for unraveling clinically meaningful subgroups in ASD. This, in turn, can be used for predicting likely responsiveness (and non-responsiveness) to specific treatment pathways. This “precision” approach to assessment and intervention will ensure that resources for appropriate intervention and supports are allocated in an evidence-based manner. This is critical as without timely recognition of the variability in the clinical presentation, neurocognitive level of functioning, and psychosocial circumstances coupled with individualized intervention, children and their families may miss key opportunities of brain plasticity available in the critical early years. ML techniques as utilized in this study offer a viable solution to address this by allowing matching interventions and supports that are tailored to the individual profile and needs.

## Data Availability Statement

The datasets presented in this study can be found in online repositories. The names of the repository/repositories and accession number(s) can be found at: https://figshare.com/articles/dataset/Autism_gene_expression_data/14251328.

## Ethics Statement

The studies involving human participants were reviewed and approved by Research Ethics Committee of the National Taiwan University Hospital. Written informed consent to participate in this study was provided by the participants' legal guardian/next of kin.

## Author Contributions

P-IL and MM carried out the statistical analysis. P-IL and VE conceived of the study and drafted the manuscript. SG participated in the study design and coordination. All authors read and approved the final manuscript.

## Conflict of Interest

The authors declare that the research was conducted in the absence of any commercial or financial relationships that could be construed as a potential conflict of interest.

## Supplementary Material

Supplementary Figure 1 The evaluation of potential batch effect due to the microarrays timing. **(A)** The kernel density distributions of gene expression levels of the two batches are shown. **(B)** Time 1 and time 2 indicate the association test results that adjusted for the time (i.e., batch) vs. the results without adjusting for the time.

Supplementary Figure 2 Randomly selected four probes associated with SCQ scores stratified by the presence of language impairment. The red and blue curves represent the group without language impairment and the group with language impairment, respectively.
