# Supplementary figures and images for: Identifying Subgroups of Patients With Autism by Gene Expression Profiles Using Machine Learning Algorithms
Source: Front Psychiatry. 2021 May 12;12:637022. doi: 10.3389/fpsyt.2021.637022 (PMC8149626; doi:10.3389/fpsyt.2021.637022)

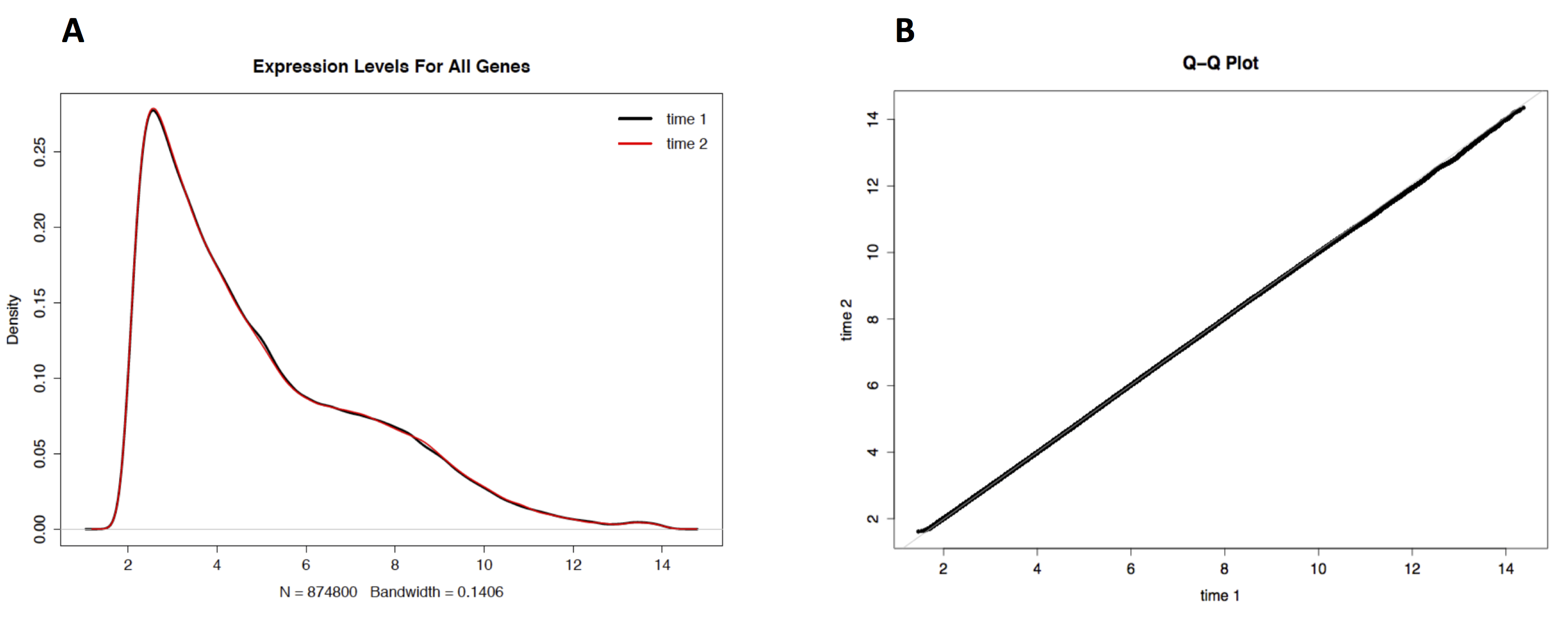

Supplement: Supplementary Figure 1 — The evaluation of potential batch effect due to the microarrays timing. (A) The kernel density distributions of gene expression levels of the two batches are shown. (B) Time 1 and time 2 indicate the association test results that adjusted for the time (i.e., batch) vs. the results without adjusting for the time. [file Image_1.TIFF]

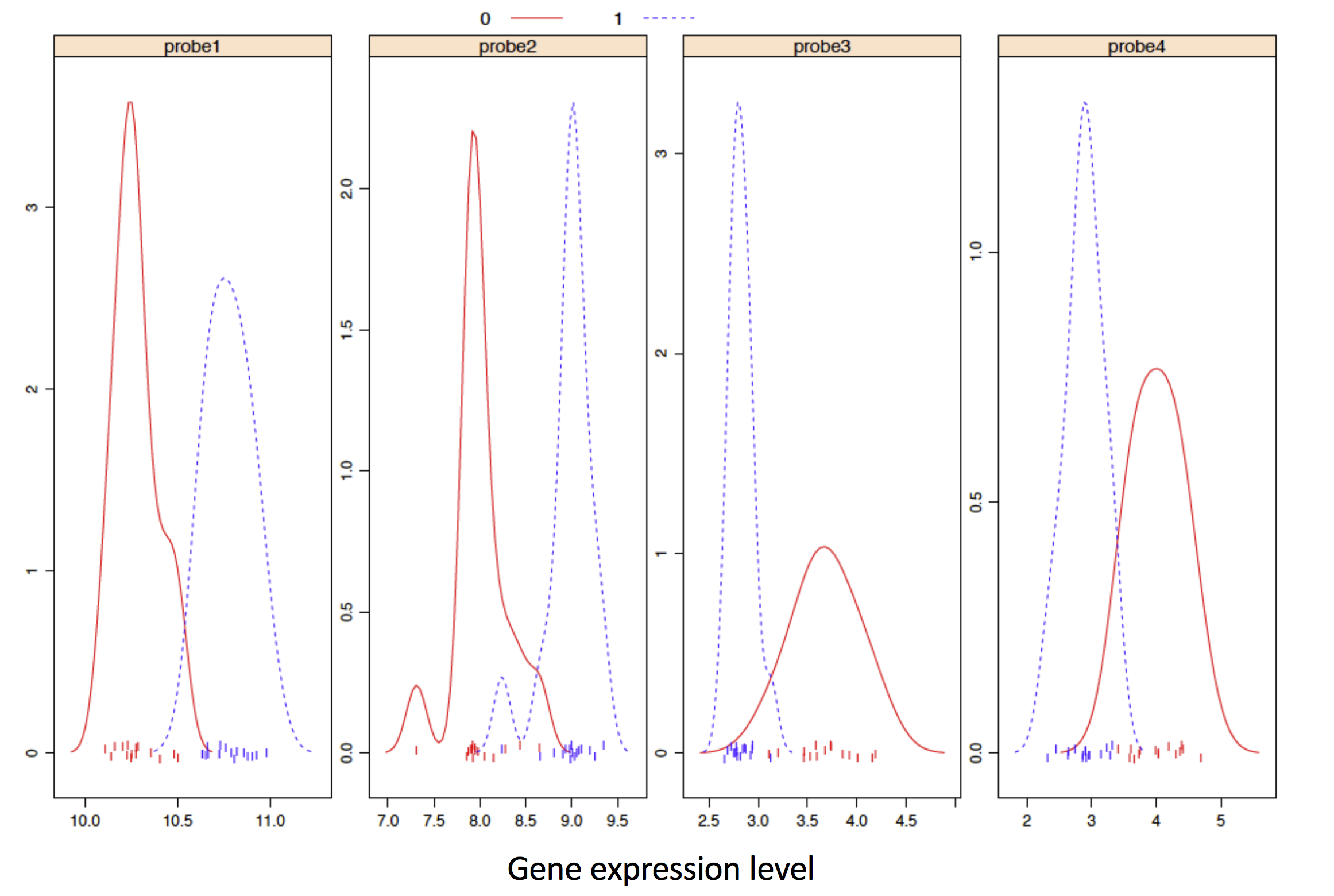

Supplement: Supplementary Figure 2 — Randomly selected four probes associated with SCQ scores stratified by the presence of language impairment. The red and blue curves represent the group without language impairment and the group with language impairment, respectively. [file Image_2.TIFF]
